# Adenocarcinoma of the lung: from BAC to the future

**DOI:** 10.1186/s13244-020-00875-6

**Published:** 2020-05-19

**Authors:** Gerard Lambe, Michael Durand, Anne Buckley, Siobhan Nicholson, Ronan McDermott

**Affiliations:** St James’s Hospital, Dublin, 8 Ireland

**Keywords:** Lung adenocarcinoma, Pathology, Computed tomography, Subsolid nodules, Solitary pulmonary nodule

## Abstract

Adenocarcinoma in situ, minimally invasive adenocarcinoma, lepidic predominant adenocarcinoma and invasive mucinous adenocarcinoma are relatively new classification entities which replace the now retired term, bronchoalveolar carcinoma (BAC). The radiographic appearance of these lesions ranges from pure, ground glass nodules to large, solid masses. A thorough understanding of the new classification is essential to radiologists who work with MDT colleagues to provide accurate staging and treatment. A 2-year review was performed of all surgically resected cases of adenocarcinoma in situ, minimally invasive adenocarcinoma and lepidic predominant adenocarcinoma in our institution. Cases are broken down by age, gender, tumour type and tumour location. A pictorial review is presented to illustrate the radiologic and pathologic features of each entity.

## Key points

1. To familiarise radiologists with the new pathological classification of adenocarcinoma of the lung.
2. To illustrate the varying CT appearances of adenocarcinomas and describe the CT features which correlate with histologic features of invasive growth.
3. To highlight how the new classification more accurately reflects the correlation between radiology, pathology and prognosis.

## The artist formerly known as BAC

A new, international, histopathologic classification of adenocarcinoma of the lung was introduced in 2011 by the International Association for the Study of Lung Cancer (IASLC), American Thoracic Society (ATS) and European Respiratory Society (ERS) [1].

Adenocarcinoma is the most common histologic type of lung cancer and accounts for over 40% of non-small cell lung cancers [2].

However, the terminology used to describe it has been inconsistent and non-uniform. For the radiologist, the new classification required some adjustment. The most striking change was the discontinuation of the term bronchoalveolar carcinoma (BAC).

The first descriptions of this entity date back to the late 19th and early 20th centuries when Malassez [3] and Musser [4] referred to lung tumours with features of BAC as ‘epithelioma’ and ‘primary cancer of the lung’ respectively. The histological hallmark of these tumours was a prominent bronchioalveolar pattern with variable extension into surrounding tissues.

Subsequent publications described tumours with similar features under a variety of names including ‘alveolar cell tumour of lung’, ‘pulmonary adenomatosis’ and ‘mucocellular papillary adenocarcinoma of the lung’ [5–8].

In 1960, Liebow [9] identified BAC as a well-differentiated adenocarcinoma and defined distinct growth patterns:

1. Single nodule
2. Disseminated nodules
3. Diffuse

In contrast to some of his predecessors, Liebow described a potential for pleural invasion, nodal metastases and distant metastases. However, the tumours were considered characteristically slow-growing, particularly the single nodule subtype.

From a pathological standpoint, 3 macroscopic subtypes of BAC were identified [10]:

1. Localised. A well circumscribed tumour, usually peripherally located, without necrosis or haemorrhage
2. Multinodular. Miliary nodules involving one or more lobes
3. Diffuse. An appearance that mimics a pneumonic process, often multilobar

A new classification system was introduced to provide more uniform terminology and diagnostic criteria. Four new entities were proposed: adenocarcinoma in situ (AIS), minimally invasive adenocarcinoma (MIA), lepidic predominant adenocarcinoma and invasive mucinous adenocarcinoma.

The aim was to separate tumours with a 100% (AIS) [11] and near 100% (MIA) [12] 5-year survival after surgical resection from those with a poor prognosis.

### Adenocarcinoma in situ

Adenocarcinoma in situ is defined as a tumour of ≤ 3 cm with pure lepidic growth but no lymphatic, vascular or pleural invasion and no tumour necrosis. The word lepidic means ‘scaly’ and is used to describe the growth of bland, pneumocytic-type tumour cells along alveoli without lymphovascular invasion.

The typical CT appearance is a ground glass nodule, although part-solid lesions are common as well as lesions with bubble-like internal lucencies.

### Minimally invasive adenocarcinoma

Minimally invasive adenocarcinoma is defined as a tumour of ≤ 3 cm with either pure lepidic growth or predominant lepidic growth and ≤ 5 mm of stromal invasion. There is no lymphatic, vascular or pleural invasion and no tumour necrosis. There is 98% overall survival after surgical resection [12].

The typical CT appearance is a ground glass nodule with a small, central solid component (≤5 mm). When adenocarcinoma of the lung includes invasive and non-invasive components, the invasive component is characteristically solid on CT and the non-invasive component is characteristically hazy and ground glass [13, 14]. The shape of a ground glass nodule does not appear to be helpful in differentiating between AIS and MIA [15].

### Lepidic predominant adenocarcinoma

Lepidic predominant adenocarcinoma is defined as a tumour of > 3 cm in total size and/or has > 5 mm lymphatic, vascular or pleural invasion with a non-mucinous lepidic predominant growth pattern.

The CT appearance is variable but the most typical appearance is a part-solid nodule or mass.

It is recommended that all invasive tumours which were previously known as non-mucinous adenocarcinomas are now described histopathologically according to the predominant subtype [16]:

*Lepidic:* As discussed above

*Acinar:* Invasive tumour composed of acini and tubules with columnar or cuboidal cells that resemble bronchial-lining epithelial cells

*Papillary:* Invasive tumour arranged as papillae structures with a fibrovascular core and complicated secondary and tertiary branches

*Micropapillary:* Small papillary tufts containing tumour cells with peripheral nuclei but without a fibrovascular core

Lepidic predominant adenocarcinoma has an excellent 5-year survival of 90% after surgical resection [1]. On the contrary, the 5-year survival after surgical resection for the micropapillary subtype is 54% compared with 81% for the non-micropapillary subtypes [17].

### Invasive mucinous adenocarcinoma

Invasive mucinous adenocarcinoma refers to a subtype of invasive adenocarcinomas with apical mucin and small, basally oriented nuclei [1]. These tumours are commonly multicentric, multilobar and bilateral [1].

The CT appearance of these lesions varies wildly from consolidation and air bronchograms to solid and subsolid nodules and masses with a bronchogenic distribution [18]. Both unifocal and multifocal forms of the disease show a lower lobe predominance (Table 1) [1].

**Table 1:**
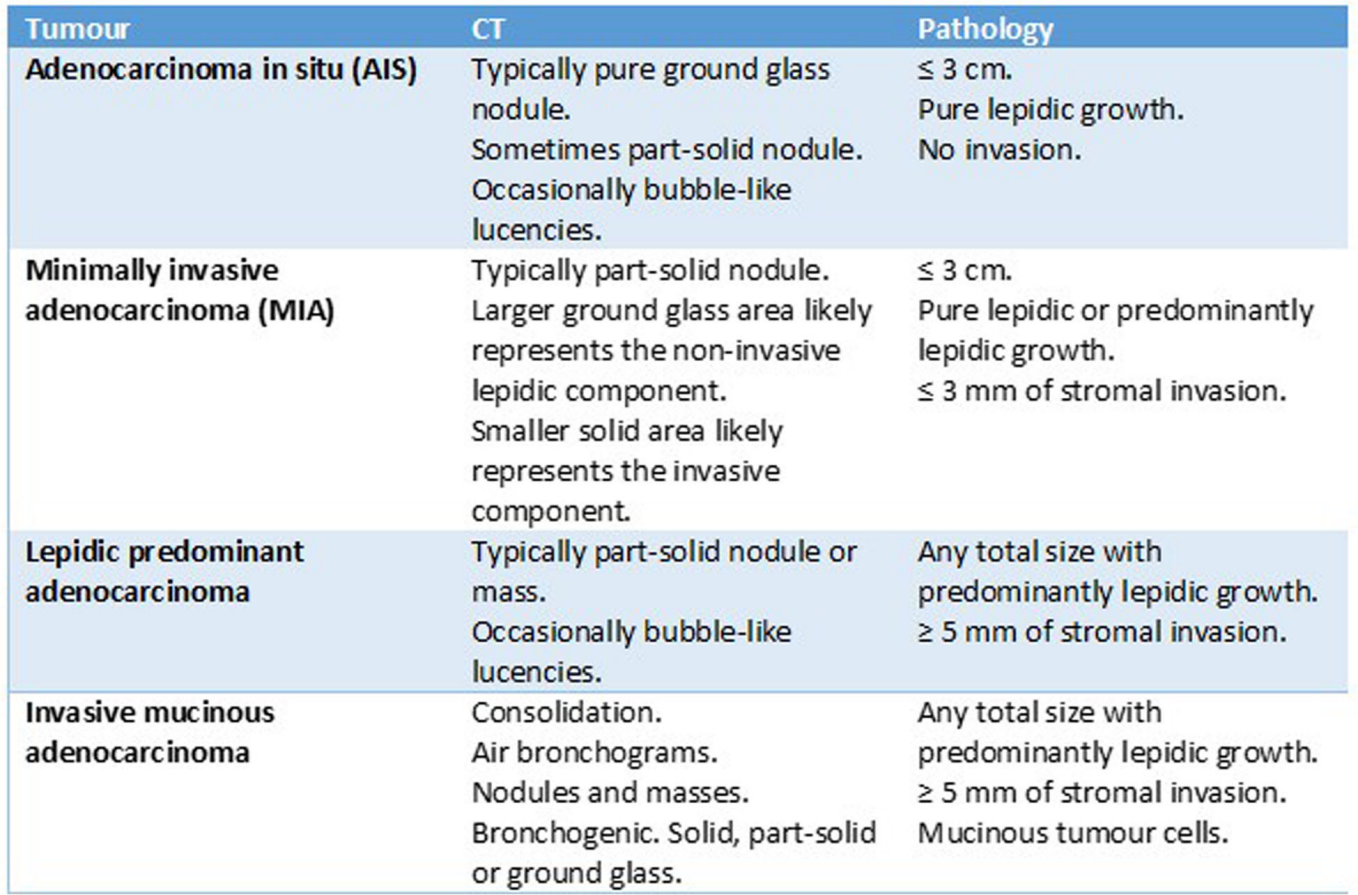
Radiological and pathological features of the lung adenocarcinoma subtypes

## A taste of our own medicine

A retrospective 2-year review was performed through our institution’s laboratory information system to identify all surgical lung resection specimens for which the final histopathological diagnosis was adenocarcinoma in situ, minimally invasive adenocarcinoma or lepidic predominant adenocarcinoma. The review covered the period January 1, 2017 to December 31, 2018.

There were 35 cases in total which included 24 females and 11 males. The age range was 44 to 86 and the mean age at pathological diagnosis was 69. The cases were divided by histologic subtype into adenocarcinoma in situ (16), minimally invasive adenocarcinoma (13) and lepidic predominant adenocarcinoma (Table 2) (6).

**Table 2:**
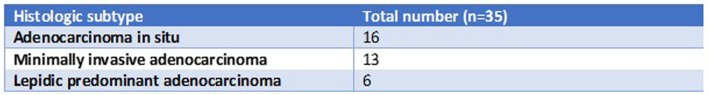
A breakdown of our cases by final histopathological diagnosis

There were 15 tumours resected from the right upper lobe, 8 from the right lower lobe, 7 from the left upper lobe and 5 from the left lower lobe.

The prior CT scans were reviewed in each case to the identify imaging features which may help to classify a lesion as either pre-invasive/minimally invasive or invasive. A pictorial review was created to illustrate the salient learning points for the radiologist.

### A pictorial review

Please see Figs. 1, 2, 3, 4, 5, 6, 7, 8, 9, 10, 11, 12, 13, 14, 15, 16, 17, 18, and 19.

**Fig. 1:**
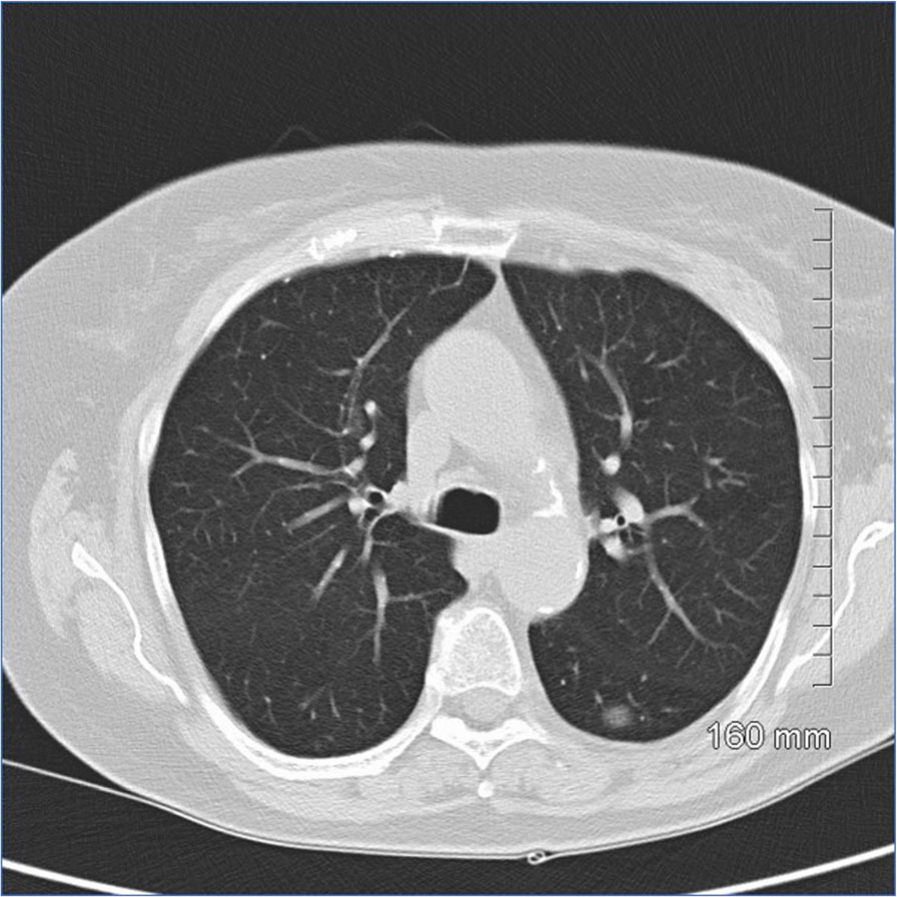
AIS on CT. A 10-mm pure ground glass nodule in the left lower lobe. Stable in size for 3 years prior to biopsy

**Fig. 2:**
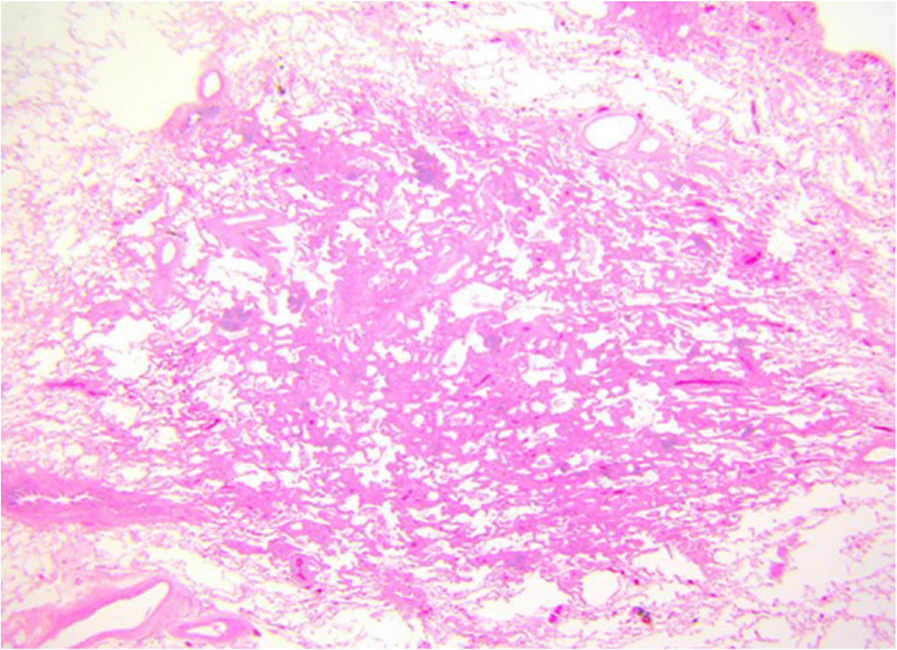
AIS histopathology: a 10-mm diameter adenocarcinoma pure lepidic growth, no invasion seen (H&E, 2 ×)

**Fig. 3:**
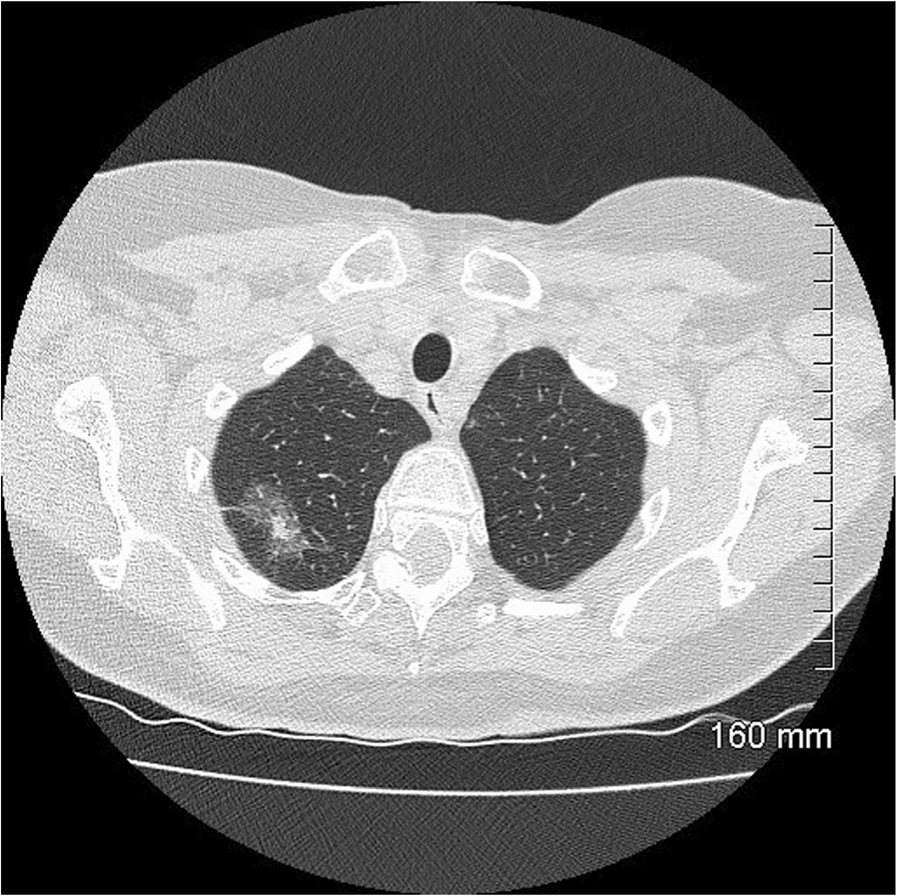
AIS on CT. A 33-mm ground glass opacity in the superior segment of the right upper lobe with a small, central solid component measuring 9 mm × 5 mm

**Fig. 4:**
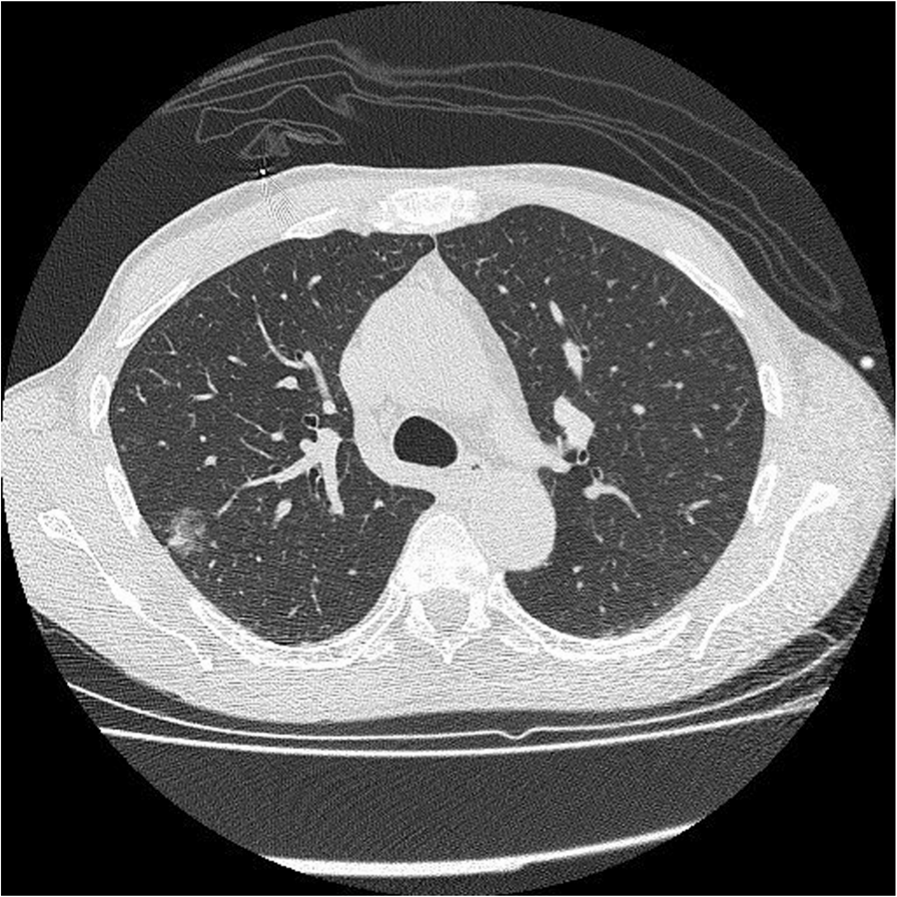
MIA on CT. A 20-mm part-solid nodule, predominantly ground glass, in the right upper lobe

**Fig. 5:**
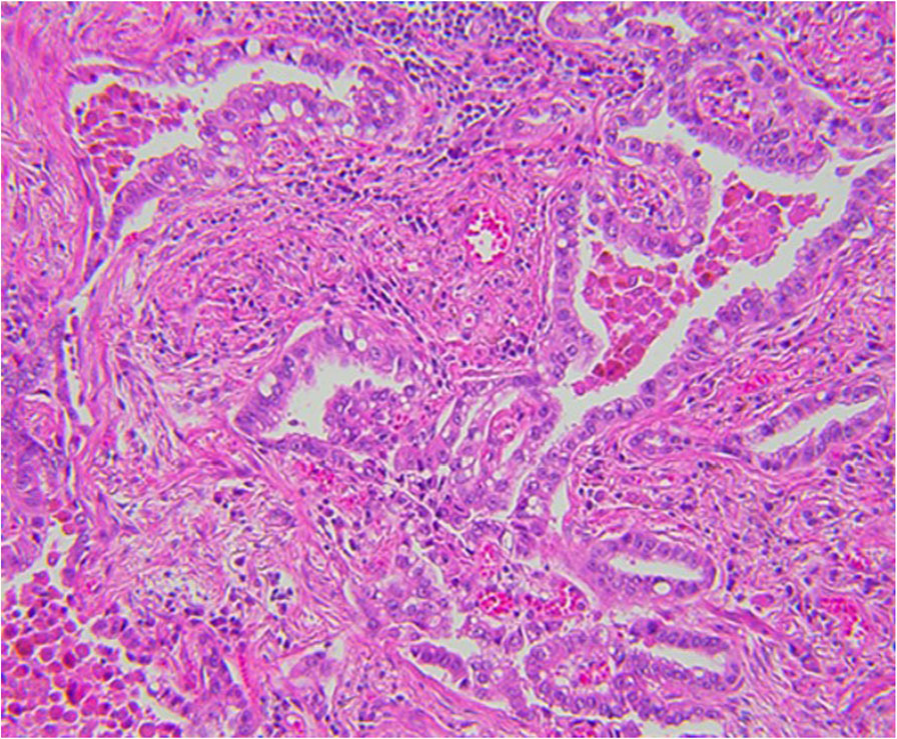
MIA histopathology: focal invasive growth < 5 mm diameter in a 17-mm lepidic pattern adenocarcinoma (H&E, 20 ×).

**Fig. 6:**
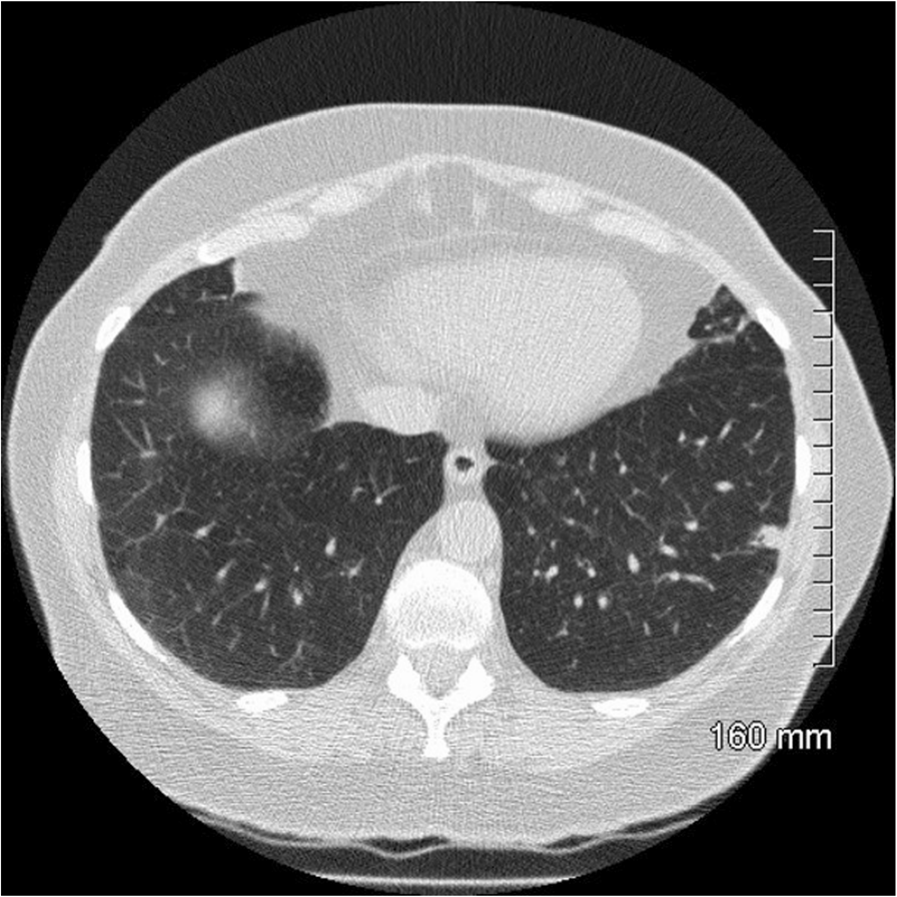
MIA on CT: 9 mm part-solid subpleural nodule, predominantly solid, in the left lower lobe

**Fig. 7:**
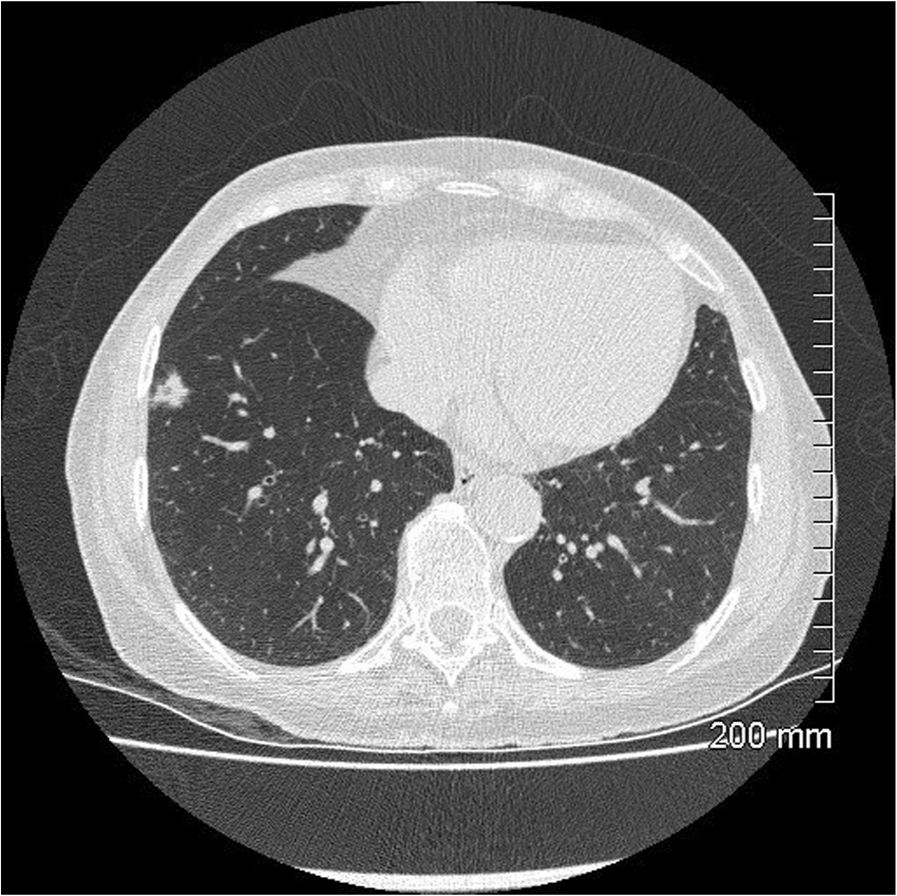
MIA on CT: 10 mm part-solid nodule, predominantly solid, in the right lower lobe

**Fig. 8:**
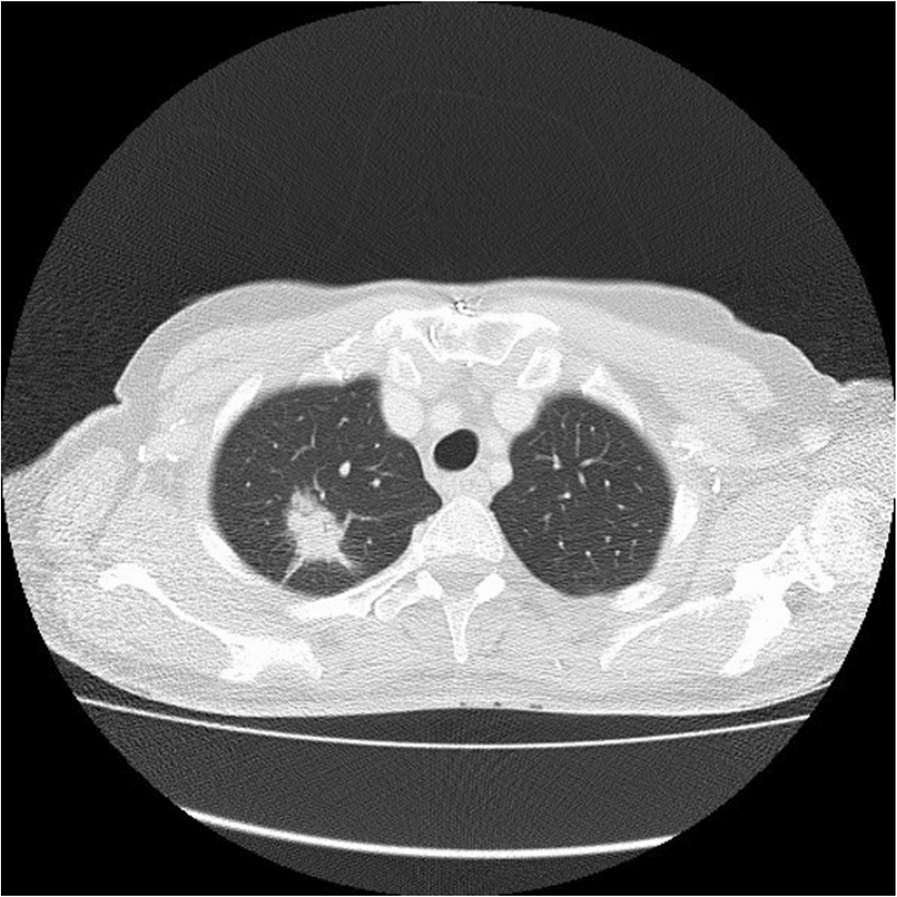
Lepidic predominant adenocarcinoma on CT: 3.2 cm predominantly solid mass in the right upper lobe with air bronchograms, thick spiculations and pleural tethering

**Fig. 9:**
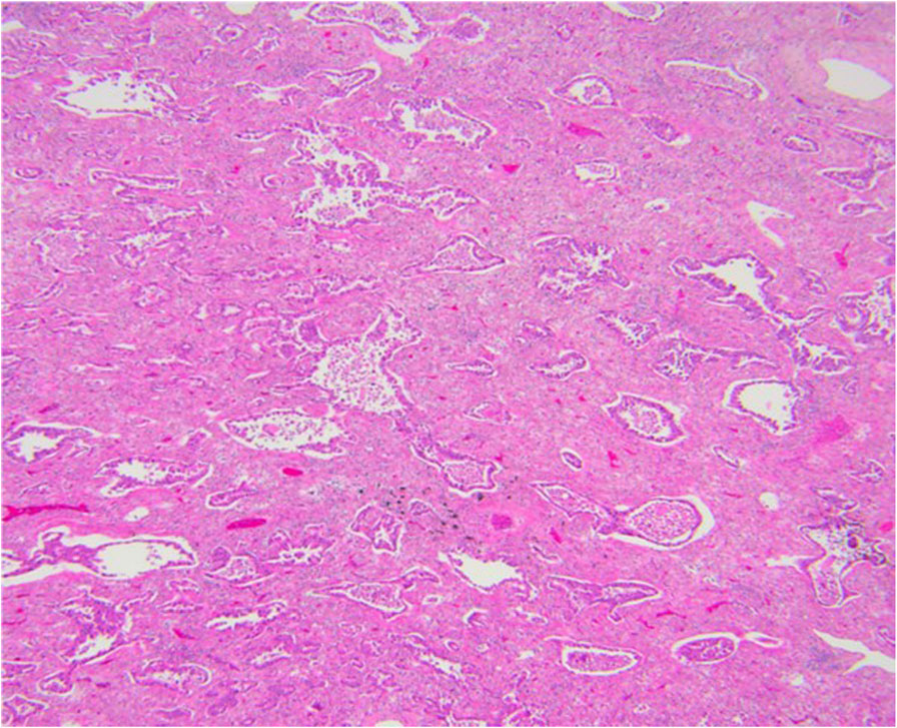
Lepidic predominant adenocarcinoma histopathology: a 25-mm maximum diameter lepidic predominant adenocarcinoma where invasive acinar growth > 5 mm (H&E, 10 ×)

**Fig. 10:**
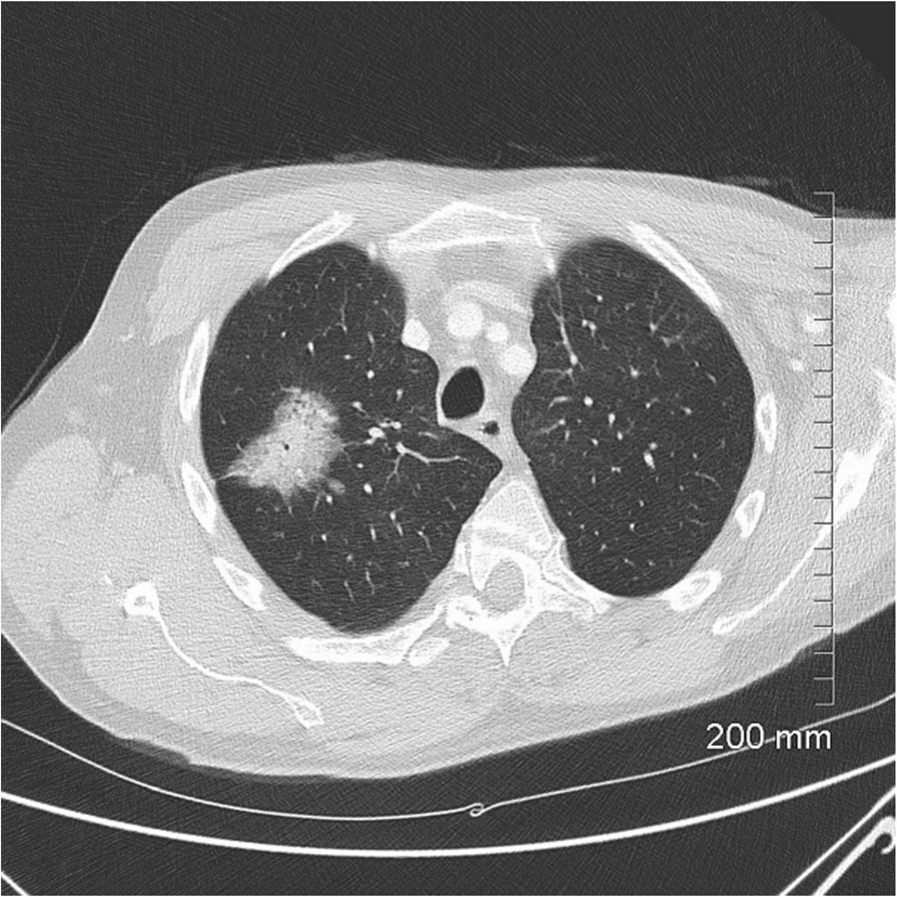
Lepidic predominant adenocarcinoma on CT. The 4.2 cm mass in the right upper lobe has spiculated margins, cystic lucencies, air bronchograms and satellite nodules

**Fig. 11:**
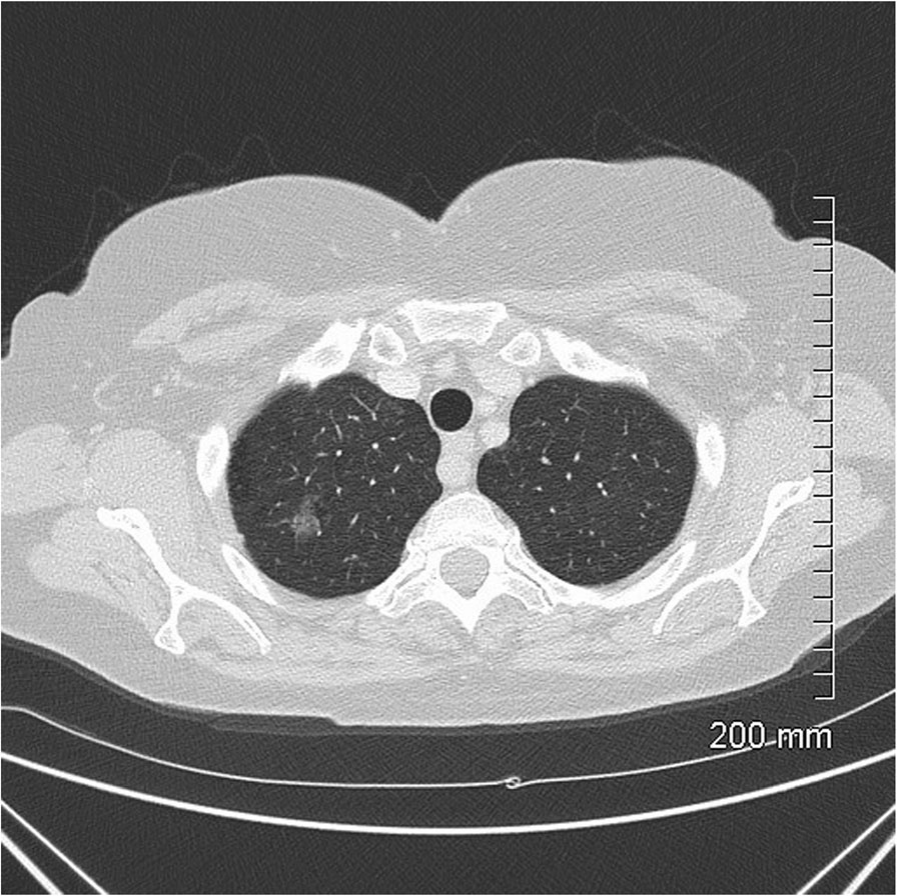
Favourable prognostic indicators: small size (< 2 cm). The pure ground glass nodule in the right upper lobe measures 10 mm. AIS

**Fig. 12:**
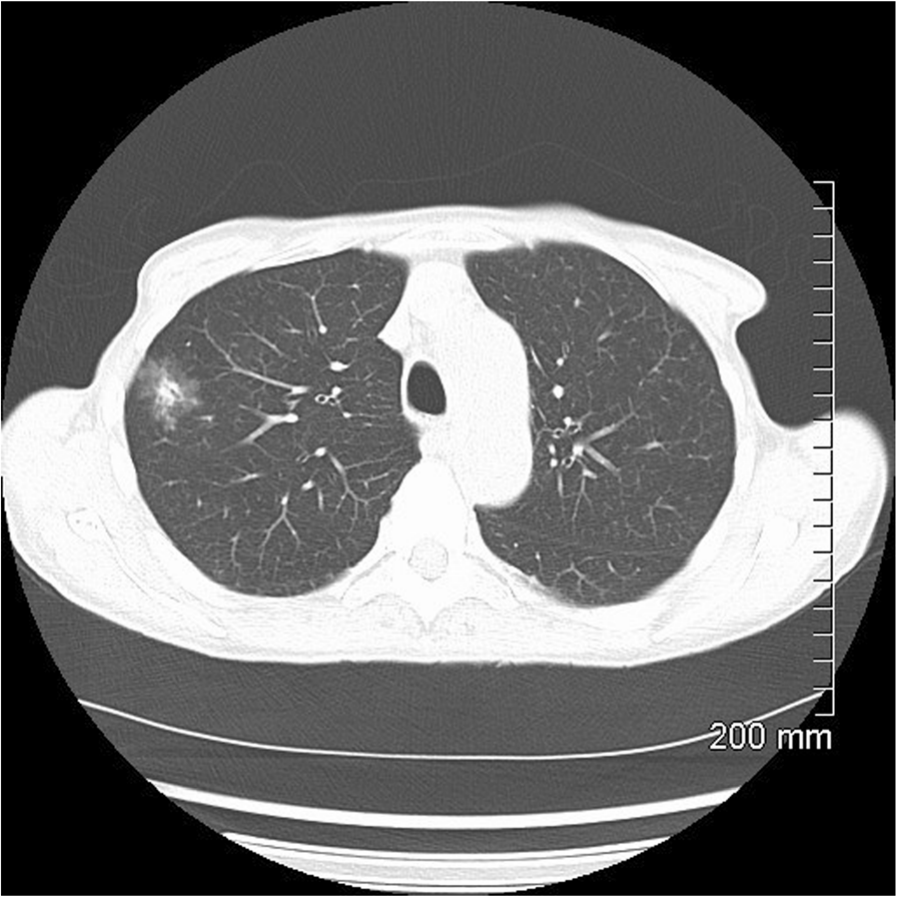
Favourable prognostic indicators: ground glass component. The part-solid nodule in the right upper lobe has a large ground glass component which correlates with lepidic growth. AIS

**Fig. 13:**
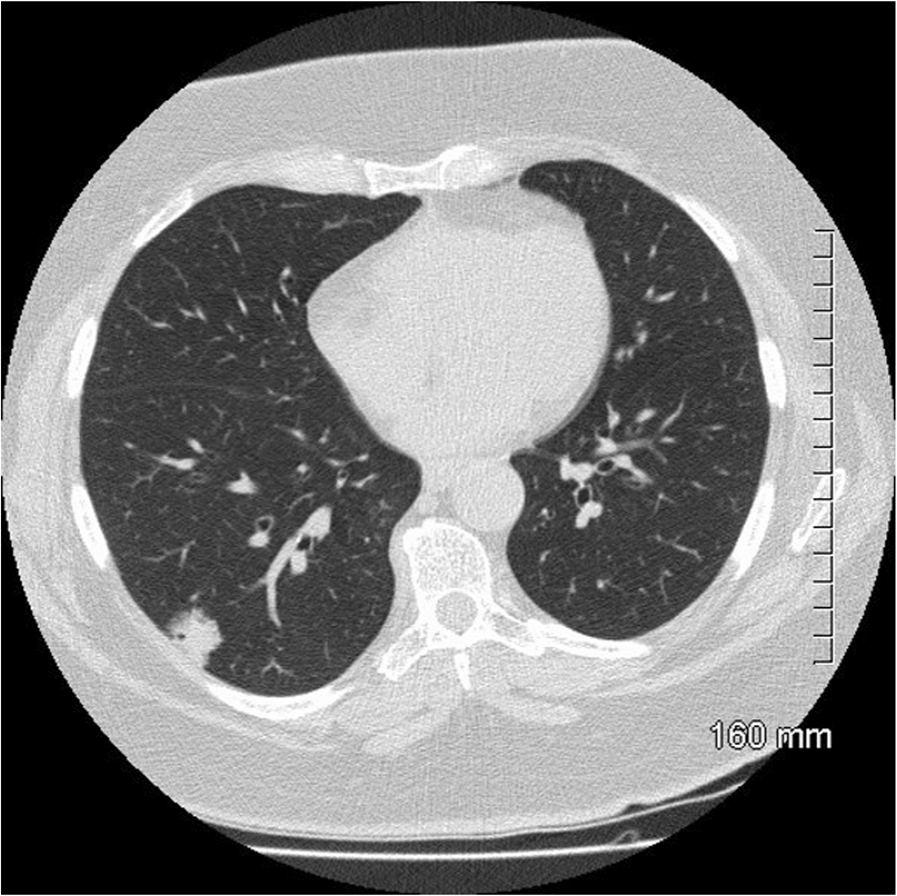
Favourable prognostic indicators: bubble-like lucencies in a part-solid right lower lobe nodule. MIA

**Fig. 14:**
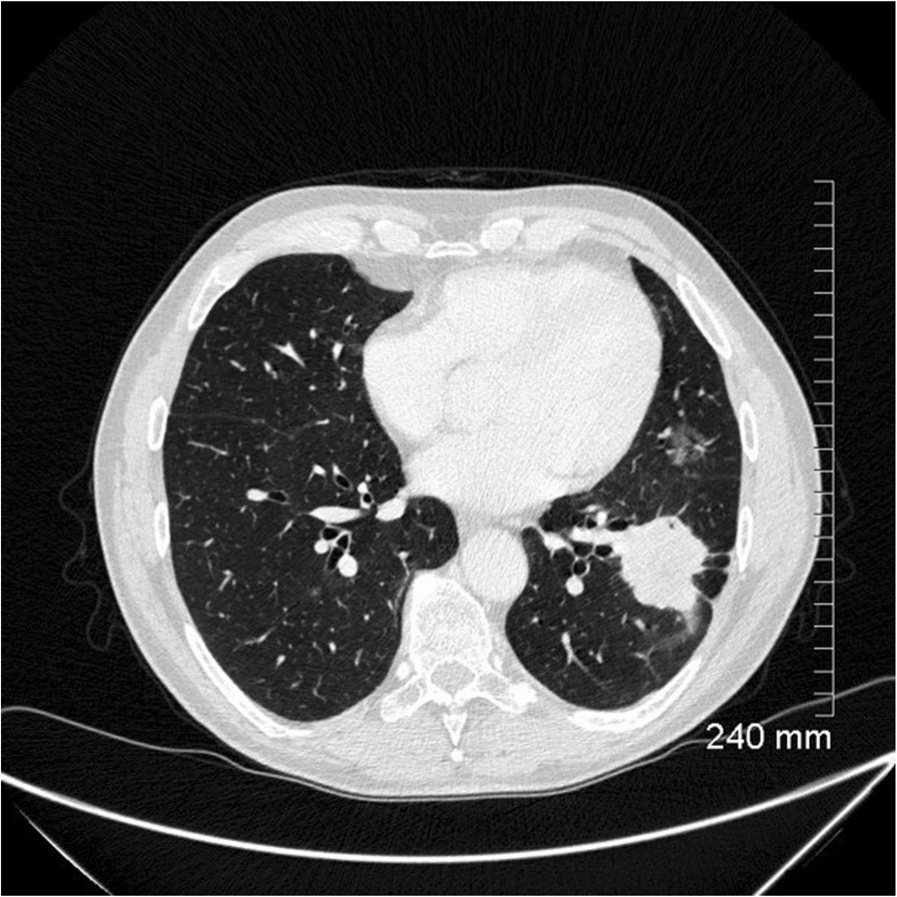
Unfavourable prognostic indicators: thickened bronchovascular bundle. The 4-cm solid mass in the left lower lobe also demonstrates spiculated margins and pleural tethering with small foci of internal cavitation. A smaller ground glass nodule in the lingula was found to represent a synchronous AIS after surgical resection

**Fig. 15:**
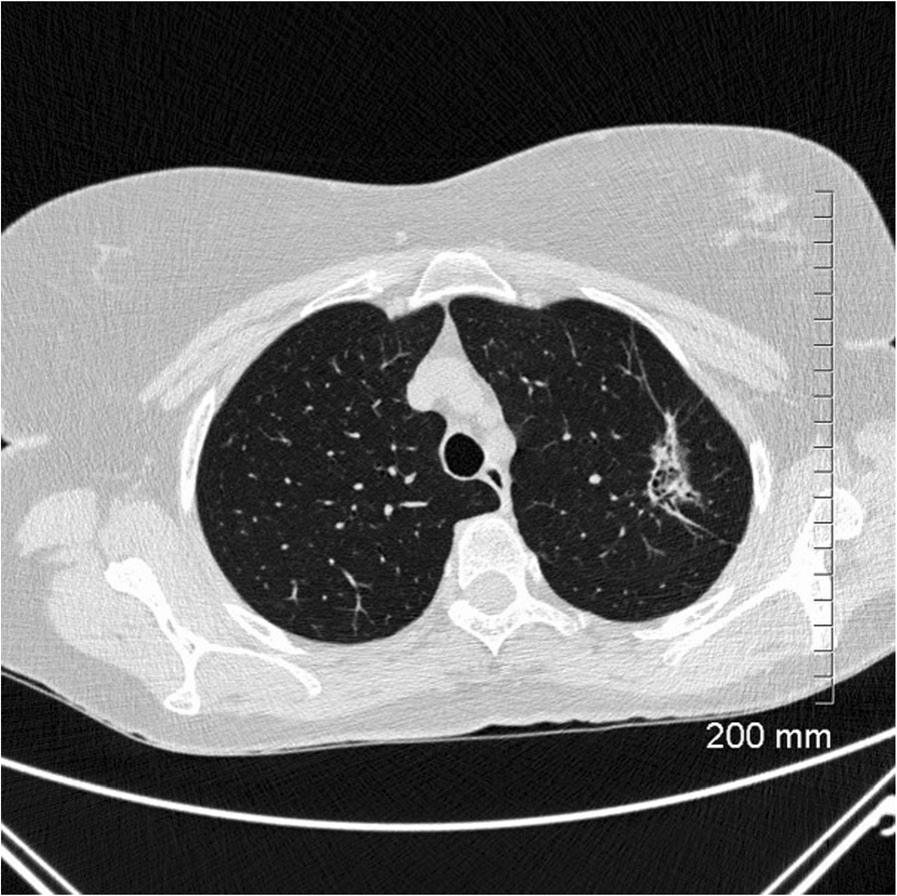
Unfavourable prognostic indicators: coarse, thick spiculations. The area of mass-like consolidation in the left upper lobe also demonstrates cystic lucencies and air bronchograms. Lepidic predominant adenocarcinoma

**Fig. 16:**
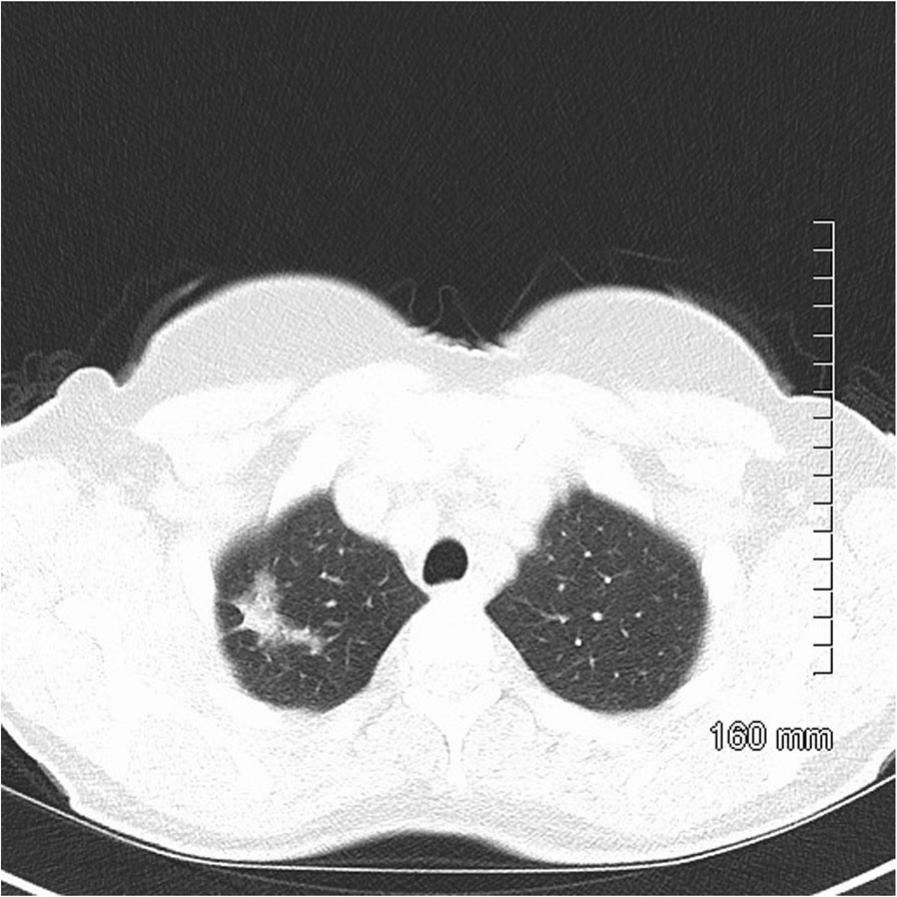
Unfavourable prognostic indicators: large size (> 2 cm). The part-solid mass at the right apex measures 3.5 cm. MIA

**Fig. 17:**
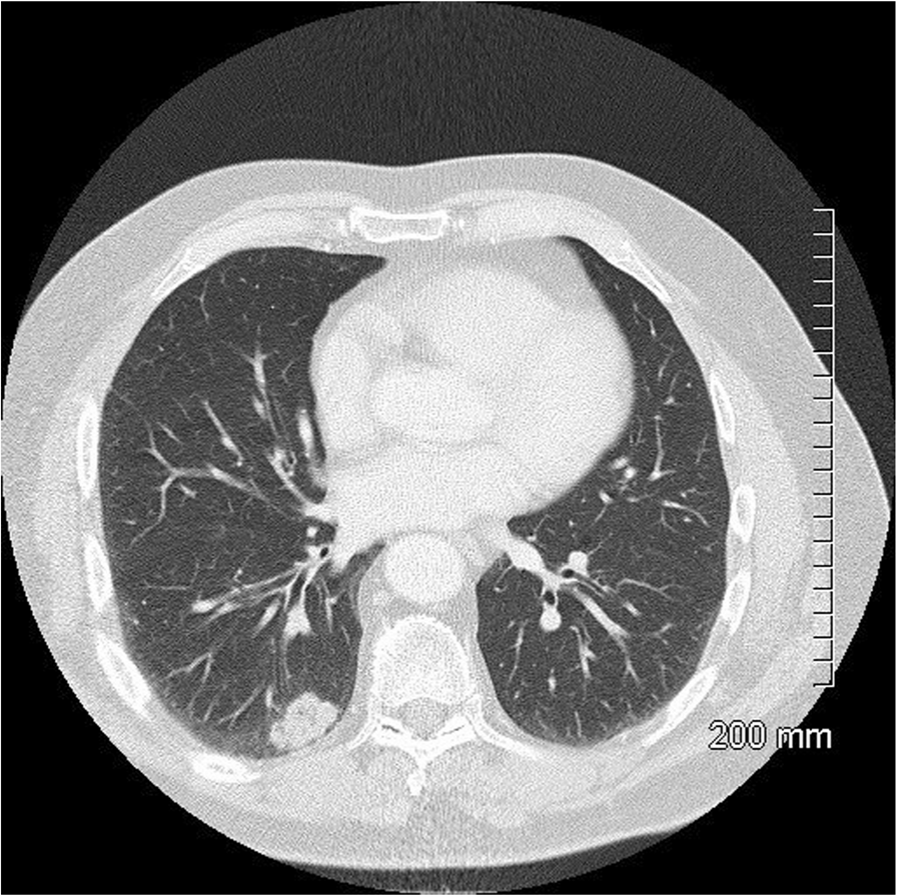
Unfavourable prognostic indicators: concave notch. The solid, lobulated subpleural mass in the right lower lobe has a concave cut or notch on its medial border. Lepidic predominant adenocarcinoma

**Fig. 18:**
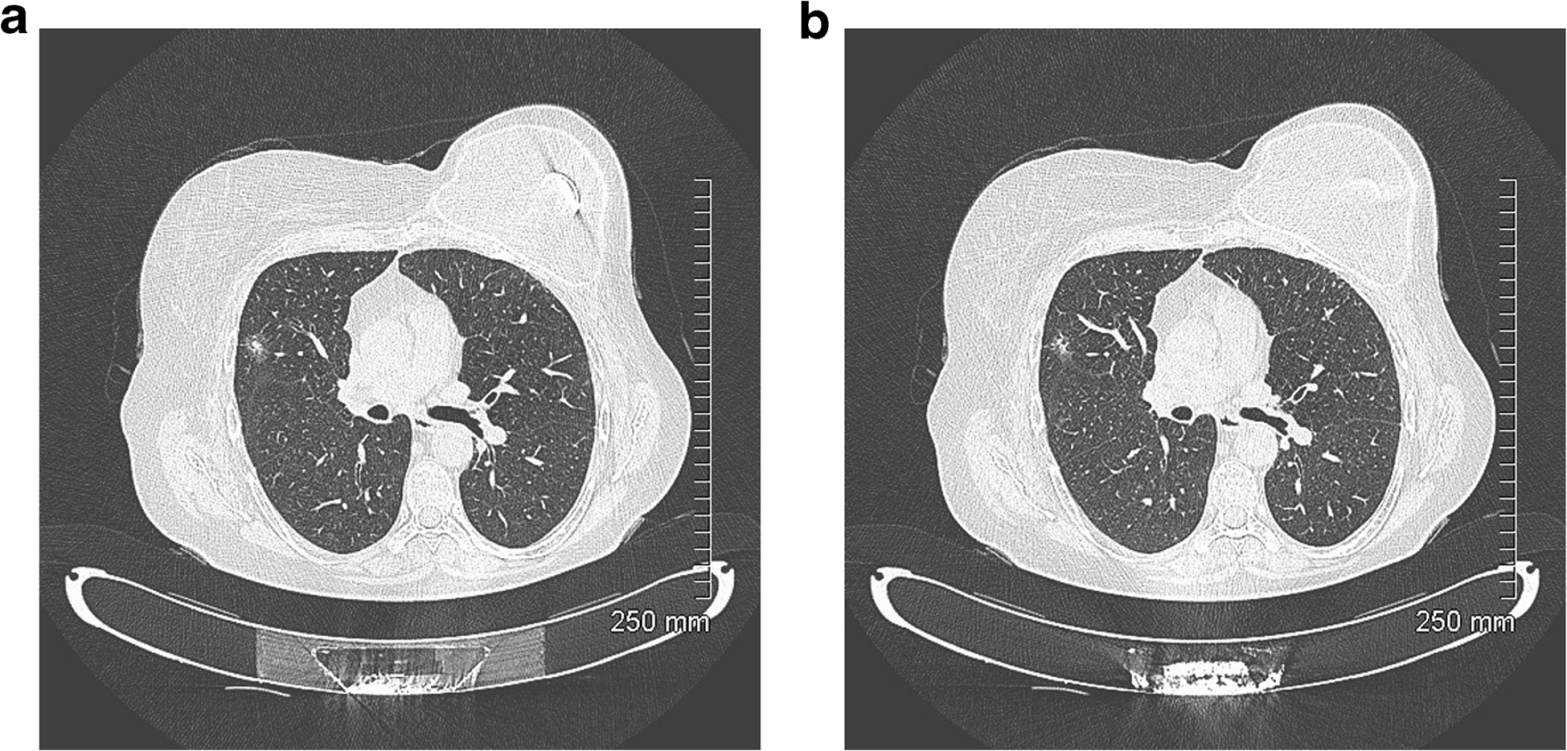
**a**, **b** Unfavourable prognostic indicators: focal pleural/fissural retraction. Part-solid nodule in the right upper lobe. Lepidic predominant adenocarcinoma

**Fig. 19:**
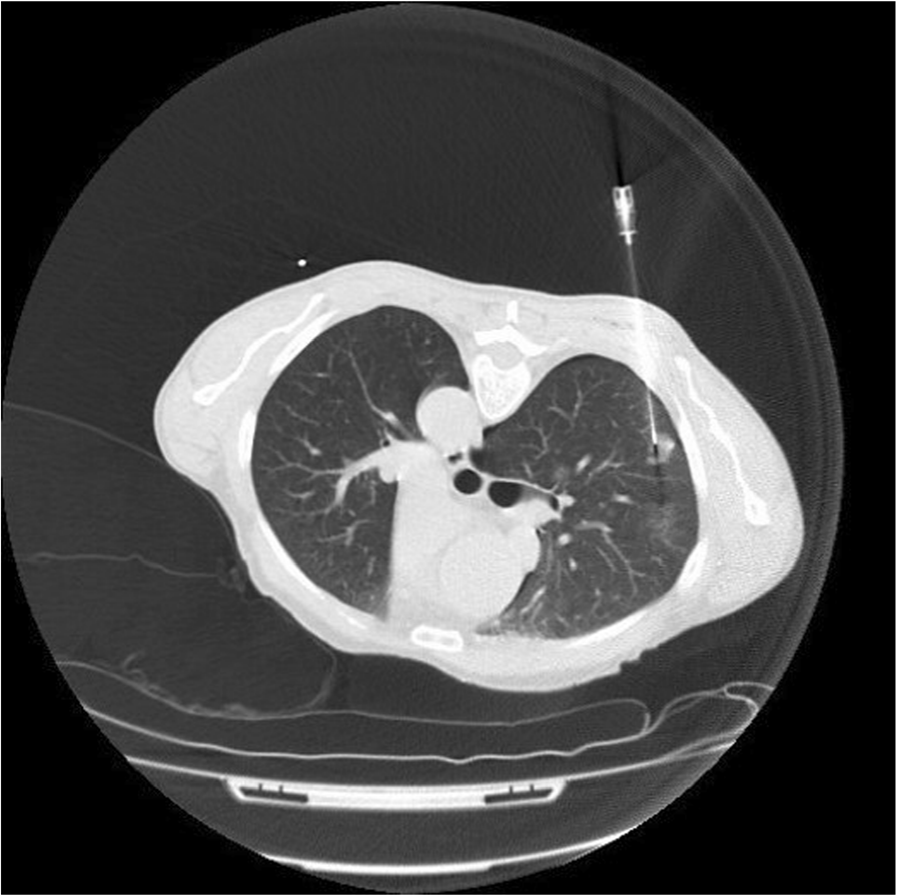
CT-guided biopsy of a part-solid nodule in the right upper lobe. Every effort should be made to target the solid component of the lesion which correlates with the invasive component of the nodule. The biopsy has been complicated by a small pneumothorax

## Imaging features and prognostication

There are a number of specific features of adenocarcinomas on CT with have been shown to represent favourable and unfavourable prognostic indicators [19].

Favourable indicators include a ground glass component, bubble-like lucencies, air bronchograms and small size. Unfavourable indicators include thick spiculations, thickened bronchovascular bundles, pleural retraction, concave cuts and large size.

Ground glass attenuation in a nodule correlates with a lepidic growth pattern in the tumour and is associated with a better prognosis [20]. Bubble-like lucencies and air bronchograms are suggestive of a well-differentiated tumour and a favourable prognosis [21, 22].

In contrast, coarse spiculations, thickened bronchovascular bundles and a low proportion of ground glass attenuation in the tumour are associated with a higher incidence of lymph node metastases and vascular invasion [23]. A smooth tumour margin and a ‘solid appearance without air bronchogram’ are suggestive of a poorly differentiated tumour and a poorer prognosis [22]. The presence of a concave cut or notch in the margin of the tumour is also considered an unfavourable prognostic indicator [24].

The size of a pulmonary nodule has prognostic relevance. For a nodule under 2 cm, the size of the lesion correlates with the incidence of vascular invasion [1]. For a nodule or mass over 3 cm, the size of the lesion correlates with the incidence of central nervous system metastases [25].

However, there is growing evidence that the solid component of a part-solid nodule is more important than the overall nodule size in staging and prognosis. It has been suggested that a solid component under 3–5 mm can exclude invasive adenocarcinoma, whereas a solid component over 9 mm has 100% specificity for the diagnosis of invasive adenocarcinoma [26, 27].

A larger solid component has more recently been associated with a shorter tumour doubling time, an increased incidence of lymph node metastases and vascular invasion and an increased incidence of local recurrence [23].

While a more detailed discussion of nodule measurement and follow-up is beyond the scope of this review, suffice is to say that measurement of the solid component of a part-solid nodule rather than the whole nodule is under consideration for the forthcoming IASLC classification. The relative proportion of the solid component in the nodule has also been shown to have prognostic value [28].

## Image-guided biopsy

Our review was based on patients in whom a final histopathological diagnosis was made from surgical resection specimens. However, it is worth noting that only 2 of 35 patients in our cohort proceeded directly to surgical resection without a pre-operative biopsy. The remaining 33 patients underwent a CT-guided biopsy to confirm the histopathological diagnosis before resection.

CT-guided biopsies were performed in 8 different centres. One to 3 core biopsies were taken from each patient using 18–20 gauge core biopsy needles.

The biopsy was complicated by a pneumothorax in 10 of 33 patients. Three of these patients required chest drain insertion. The remaining 7 patients required no treatment and were followed up radiologically to resolution.

Thirteen patients had pulmonary haemorrhage which presented as a focal opacity on CT. Two patients had post-procedural haemoptysis and both were managed conservatively (Table 3).

**Table 3:**
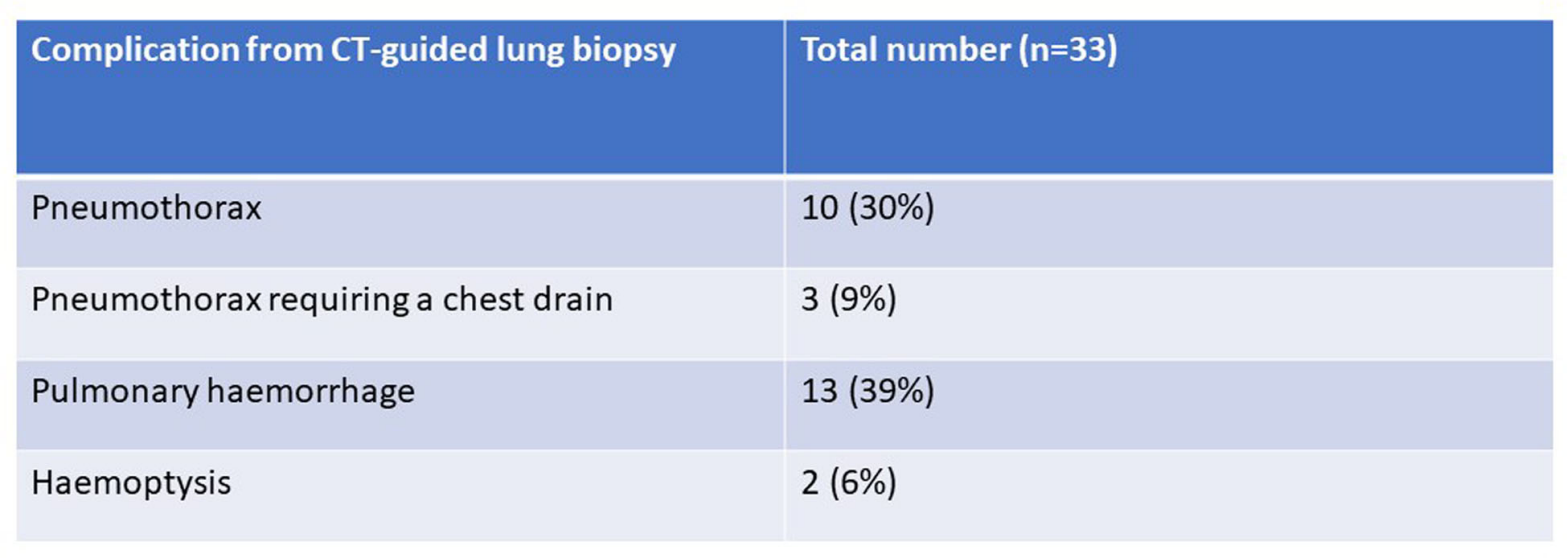
Complications from CT-guided lung biopsies

There are two special considerations to be made when contemplating biopsy of a primary lung adenocarcinoma. Every effort should be made to target the solid component of any part-solid nodule, as it is the solid component that represents invasive growth and the ground glass component that represents lepidic growth. Failure to sample the solid component may falsely identify an invasive lesion as a pre-invasive or minimally invasive lesion.

Secondly, every effort should be made to obtain enough tissue for both morphological and molecular/immunohistochemical analysis. This allows testing for mutations, including EGFR and KRAS, which may determine suitability for tyrosine kinase inhibitor therapy. Traditionally, core biopsy has been considered superior to fine needle aspiration (FNA) for morphological analysis but some studies have found FNA to be superior. Rekhtman et al. for example, found FNA cell blocks to be adequate for molecular analysis in 98% of samples [29]. They suggested that the number of tumour cells in a core biopsy may be inadequate due to the large number of stromal cells. By contrast, samples obtained by FNA tend to have a relatively higher number of tumour cells. It is thus useful, where resources allow, to have a cytotechnologist present at the time of the FNA or biopsy to confirm the adequacy of the sample.

## Conclusion

There is a new classification system for adenocarcinoma of the lung which has been designed to more accurately reflect the correlation between radiology, pathology and prognosis. The classification describes a spectrum which includes pre-invasive, minimally invasive and invasive lesions. The pre-invasive and minimally invasive lesions have almost 100% survival after surgical resection.

Diagnosis of these lesions is multidisciplinary, but primarily histopathological. The imaging features of lesions on this spectrum vary wildly with a significant amount of overlap. However, the take home message for the radiologist is that certain imaging features, including the size, shape and attenuation of the lesion, will help to determine on which side of the spectrum the lesion is likely to fall. Correct interpretation of the imaging features will help in accurate diagnosis and prognostication.

## Data Availability

All data generated or analysed during this study are included in this published article

## Abbreviations

AAH: Atypical adenomatous hyperplasia
AIS: Adenocarcinoma in situ
ATS: American Thoracic Society
BAC: Bronchoalveolar carcinoma
EGFR: Epidermal growth factor receptor
ERS: European Respiratory Society
IASLC: International Association for the Study of Lung Cancer
KRAS: Kirsten rat sarcoma viral oncogene homolog
MIA: Minimally invasive adenocarcinoma

## References

[1] Travis WD, Garg K, Franklin WA (2005). Evolving concepts in the pathology and computed tomography imaging of lung adenocarcinoma and bronchioloalveolar carcinoma. J Clin Oncol.

[2] Travis WD, Travis LB, Devesa SS (1995). Lung cancer. Cancer.

[3] Malassez L (1876) Examen histologique d’un cas de cancer encephaloide du poumon (epithelioma). Arch Physiol Norm Pathol 1876;3:353-72 [Google Scholar].

[4] Musser JH (1903) Primary cancer of the lung. U Penn Med Bull 1903:16:289-96 [Google Scholar].

[5] Sweany HC (1935) A so-called alveolar cell cancer of the lung. Arch Pathol 1935;19:203-7 [Google Scholar].

[6] Wood DA, Pierson PH (1945) Pulmonary alveolar adenomatosis in man. Is this the same disease as jaagsiekte in sheep? Am Rev Tuberc 1945;51:205-24 [Google Scholar].

[7] Osserman KE, Neuhof H (1950). Mucocellular papillary adenocarcinoma of the lung, lobectomy, five-year follow-up. J Thorac Surg.

[8] Gandara David R., Aberle Denise, Lau Derick, Jett James, Akhurst Tim, Mulshine James, Berg Christine, Patz Edward F. (2006). Radiographic Imaging of Bronchioloalveolar Carcinoma: Screening, Patterns of Presentation and Response Assessment. Journal of Thoracic Oncology.

[9] Liebow AA (1960). Bronchiolo-alveolar carcinoma. Adv Intern Med.

[10] Weissferdt A, Moran CA (2014) Reclassification of early stage pulmonary adenocarcinoma and its consequences. J Thorac Dis 6(5):S581-S588–S588. 10.3978/j.issn.2072-1439.2014.07.41 PMC4209387 25349709

[11] Sakurai H, Dobashi Y, Mizutani E (2004). Bronchioloalveolar carcinoma of the lung 3 centimeters or less in diameter: a prognostic assessment. Ann Thorac Surg.

[12] Tsuta K, Kawago M, Inoue E (2013). The utility of the proposed IASLC/ATS/ERS lung adenocarcinoma subtypes for disease prognosis and correlation of driver gene alterations. Lung Cancer.

[13] Suzuki K, Kusumoto M, Watanabe S, Tsuchiya R, Asamura H (2006). Radiologic classification of small adenocarcinoma of the lung: radiologic-pathologic correlation and its prognostic impact. Ann Thorac Surg.

[14] Asamura H, Suzuki K, Watanabe S, Matsuno Y, Maeshima A, Tsuchiya R (2003). A clinicopathological study of resected subcentimeter lung cancers: a favorable prognosis for ground glass opacity lesions. Ann Thorac Surg.

[15] Kim HY, Shim YM, Lee KS, Han J, Yi CA, Kim YK (2007). Persistent pulmonary nodular ground-glass opacity at thin-section CT: histopathologic comparisons. Radiology.

[16] Solis LM, Behrens C, Raso MG (2012). Histologic patterns and molecular characteristics of lung adenocarcinoma associated with clinical outcome. Cancer.

[17] Makimoto Y, Nabeshima K, Iwasaki H (2005). Micropapillary pattern: a distinct pathological marker to subclassify tumours with a significantly poor prognosis within small peripheral lung adenocarcinoma (</=20 mm) with mixed bronchioloalveolar and invasive subtypes (Noguchi’s type C tumours). Histopathology.

[18] Wislez M, Massiani M-A, Milleron B (2003). Clinical characteristics of pneumonic-type adenocarcinoma of the lung. Chest.

[19] Gaikwad Anand, Gupta Ashish, Hare Sam, Gomes Marcio, Sekhon Harman, Souza Carolina, Inacio Joao, Lad Shilpa, Seely Jean (2012). Primary adenocarcinoma of lung: A pictorial review of recent updates. European Journal of Radiology.

[20] Kodama K, Higashiyama M, Yokouchi H (2001). Prognostic value of ground-glass opacity found in small lung adenocarcinoma on high-resolution CT scanning. Lung Cancer.

[21] Saito H, Yamada K, Hamanaka N (2009). Initial findings and progression of lung adenocarcinoma on serial computed tomography scans. J Comput Assist Tomogr.

[22] Yabuuchi H, Murayama S, Murakami J (2000). High-resolution CT characteristics of poorly differentiated adenocarcinoma of the peripheral lung: comparison with well differentiated adenocarcinoma. Radiat Med.

[23] Aoki T, Tomoda Y, Watanabe H (2001). Peripheral lung adenocarcinoma: correlation of thin-section CT findings with histologic prognostic factors and survival. Radiology.

[24] Ikehara M, Saito H, Kondo T (2012). Comparison of thin-section CT and pathological findings in small solid-density type pulmonary adenocarcinoma: prognostic factors from CT findings. Eur J Radiol.

[25] Austin JHM, Mujoomdar A, Powell CA, Pearson GDN, Raftopoulos H (2008). Carcinoma of the lung and metastatic disease of the central nervous system. Am J Respir Crit Care Med.

[26] Cohen JG, Reymond E, Lederlin M (2015). Differentiating pre- and minimally invasive from invasive adenocarcinoma using CT-features in persistent pulmonary part-solid nodules in Caucasian patients. Eur J Radiol.

[27] Lee KH, Goo JM, Park SJ (2014). Correlation between the size of the solid component on thin-section CT and the invasive component on pathology in small lung adenocarcinomas manifesting as ground-glass nodules. J Thorac Oncol.

[28] Honda T., Kondo T., Murakami S., Saito H., Oshita F., Ito H., Tsuboi M., Nakayama H., Yokose T., Kameda Y., Isobe T., Yamada K. (2013). Radiographic and pathological analysis of small lung adenocarcinoma using the new IASLC classification. Clinical Radiology.

[29] Rekhtman N, Brandt SM, Sigel CS (2011). Suitability of thoracic cytology for new therapeutic paradigms in non-small cell lung carcinoma: high accuracy of tumor subtyping and feasibility of EGFR and KRAS molecular testing. J Thorac Oncol.

